# 10.L. Round table: Health literacy in health professionals – conceptualising and piloting a new measuring instrument

**DOI:** 10.1093/eurpub/ckac129.661

**Published:** 2022-10-25

**Authors:** Organised by: Careum Center for Health Literacy (Switzerland), Gesundheit Österreich GmbH (Austria), University of Bielefeld (Germany), Chair persons: Doris Schaeffer (Germany), Saskia De Gani (Switzerland)

## Abstract

**Background:**

Studies have shown that the population's health literacy is low in many countries, including Austria, Germany, and Switzerland. In order to improve the health literacy of patients and the population, health professionals have an essential role to play. However, as most studies have been conducted either outside of Europe or among health professionals in training, there is a lack of a clear definition, clear-cut concept, and reliable data on the professional health literacy of the healthcare workforce in Europe so far.

**Approach of the Pilot Study:**

The present study is a pilot study that aims to remedy this shortcoming. The underlying notion of health literacy is based on the definition by the HLS-EU Consortium and the HLS19-study. Following these definitions, a joint concept of professional health literacy was developed. It is comprised of the following complexes: a) communication with patients and users, b) dissemination of health related information with patients in a comprehensible manner, and c) dealing with relevant professional knowledge by health professionals. Based on this conceptual reflections, a quantitative survey was developed and conducted among health professionals in Austria, Germany, and Switzerland. This process revealed some challenges and limitations of a standardised questionnaire, as national discourses vary and different professions require distinctive vocabulary and frameworks of reference. Moreover, differences in professional qualifications and training challenge the comparability of both sample and results.

**Objectives of the Workshop:**

The objective of this workshop is threefold.

a. The first is to present the working group's concept, its experience with, and reflections on this first of its kind study in Europe - all before the backdrop of previous endeavours to examine professional health literacy. This is the more important as interest in professional and organisational health literacy is growing.

b. The second objective includes the presentation of the survey instrument, its development, and preliminary results (the analysis of the study's data will not be completed by the time of the workshop).

c. The third objective is to provide a platform for discussion about the study as well as the challenges and potential limitations of a wider international comparison across a larger variety of health professions.

**Added Value of the Workshop:**

The workshop will provide an overview of the general context, the approaches in the field of professional health literacy, and the concept. This combination offers a unique opportunity to discuss the study, its questionnaire, and preliminary results, while considering aspects and issues in this field of research in general. This discussion is essential, as it supports identifying opportunities and limitations in order to develop solutions in this field of research - and contribute to the possible progress in the development of the role of health professionals.

**Key messages:**

• Professional health literacy is essential to improve patients’ health literacy.

• This workshop discusses potentials, challenges, and potential limitations examining this field.

**Speakers/Panellists:**

**Robert Griebler**

Austrian National Public Health Institute, Vienna, Austria

**Lennert Griese**

Bielefeld University, Bielefeld, Germany

**Alexander Haarmann**

Hertie School, Berlin, Germany

**Rebecca Jaks**

Careum, Zurich, Switzerland

